# Supplementary material for: Practical Opportunities to Improve the Impact of Health Risk Assessment on Environmental and Public Health Decisions
Source: Int J Environ Res Public Health. 2022 Apr 1;19(7):4200. doi: 10.3390/ijerph19074200 (PMC8998966; doi:10.3390/ijerph19074200)
Supplement: Supplementary file 1 [file ijerph-19-04200-s001.zip › ijerph-1624355-supplementary.pdf]

Practical opportunities to improve the impact of health risk assessment on environmental and public health decisions

Supplementary material

Usage of health risk assessment in decision-making: Questionnaire and results of the first three target groups (*n* = 47)

1. **Your activity or area of interest**

		All answers	All %	Group 1	Group 2	Group 3
a.	Research	24	51.1	7	15	2
b.	Administration/regulation	10	21.3	2	0	8
c.	Public health	5	10.6	1	0	4
d.	Industrial hygiene	4	8.5	1	0	3
e.	Emergency management	1	2.1	0	0	1
f.	Toxicology	1	2.1	0	0	1
g.	Human health risk assessment	1	2.1	0	0	1
h.	Job safety analysis (JSA), permit to work (PTW), management of change (MOC)	1	2.1	0	0	1

Comments: Analysis of answers according to the responders' backgrounds counted professionals with the backgrounds in "toxicology" and "human health risk assessment" together with those with a background in "research", professionals with the background in "emergency management" together with "administration/regulation", and professional with a background in "job safety analysis" together with "industrial hygiene" backgrounds. Since groups 1 and 2 were comprised mostly by researchers it is not surprising that more than half of the responders had a background in research.

2. **Do you have any previous experience with decision-making cases that required the assessment of health risks or impacts?**

		All answers	All %	Group 1	Group 2	Group 3
a.	Yes	26	56.5	5	2	19
b.	No	20	43.5	5	13	2

3. **In your view, which types of the health risk assessment (HRA) results (endpoints) are most useful in your decision-making?**

Multiple answers possible; % shows the percentage of responders that selected the option

		All answers	All %	Group 1	Group 2	Group 3
a.	Probability (e.g., 1:10000) of developing a disease (i.e. getting ill) due to repeated (chronic) exposure to hazardous substances (e.g. at a work place)	28	59.6	2	10	16
b.	Increase of the probability of disease occurrence due to specific exposure	25	53.2	5	7	3
c.	Number of newly diseased per e.g. 100,000 persons due to the exposure to hazardous substances (incidence)	21	44.7	5	6	10
d.	Ratio of a disease occurrence between the exposed and non-exposed population	19	40.4	4	6	9
e.	Number of diseased due to acute exposure to a hazardous (e.g., carcinogenic) substance	17	36.2	2	5	10
f.	Costs related to actions/measures needed for reducing the exposure	15	31.9	1	1	13
g.	Increase of the number of diseased persons in a certain period among the entire population of interest (e.g., prevalence variation during selected time period)	15	31.9	3	4	8
h.	Additional healthcare costs due to exposure to hazardous substances	11	23.4	1	2	8
i.	Number of diseases per e.g., 100,000 persons	11	23.4	3	2	6
j.	Other (none of the above)	1	2.1	1	0	0

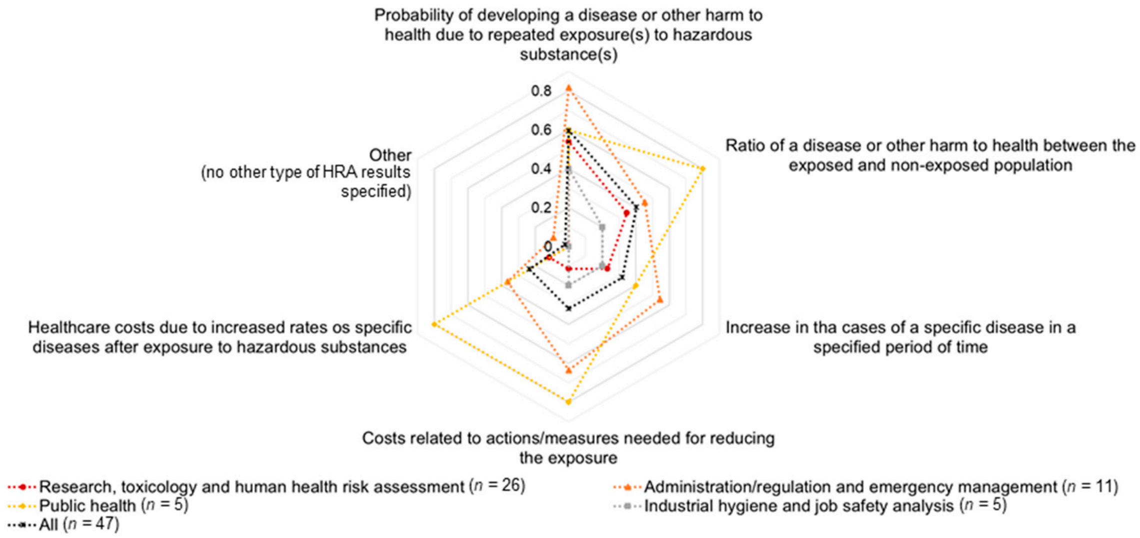

Figure S1: Most useful types of HRA results for decision making – proportions of responses from groups 1 to 3 according to the background of the responders (showing responses as in Figure 1)

4. What other information – besides the type of HRA result – is important in making a decision?

Multiple answers possible; % shows the percentage of responders that selected the option

		All answers	All %	Group 1	Group 2	Group 3
a.	HRA procedure	28	59.6	11	8	9
b.	Participants in the assessment process (multiple stakeholders)	25	53.2	5	7	13
c.	Reasons for making HRA	25	53.2	8	5	12
d.	Reputation and credibility of the risk assessor	22	46.8	3	6	13
e.	Cost of HRA procedure	16	34.0	5	3	8
f.	Users of HRA results (besides yourself)	15	31.9	3	2	10
g.	Need for HRA in a specific context	15	31.9	6	1	8
h.	Duration of the HRA procedure	15	31.9	3	6	6
i.	Commissioner of HRA	5	10.6	1	1	3
j.	Other:<br>- Assessment needs to be made in consideration of the assessment context	1	2.1	1	0	0

Comments: No clear type of additional information besides the type of HRA results that is also important in making a decision.

5. How does HRA influence your decision-making?

Multiple answers possible; % shows the percentage of responders that selected the option

		All answers	All %	Group 1	Group 2	Group 3
a.	HRA improves transparency of the decision-making process	32	68.1	9	8	15
b.	HRA results are direct and most important foundation of decision-making	26	55.3	8	8	10
c.	Until now HRA results have not been used enough, they could be used more often (regularly)	18	38.3	5	7	6
d.	Although HRA is presented as an important factor it does not have real effect on decision-making	5	10.6	3	2	0
e.	I do not know	4	8.5	0	1	4
f.	Expectations and role of HRA are too high and irrational	1	2.1	1	0	0
g.	HRA unjustifiably prolongs the decision-making, it is not needed	0	0	0	0	0
h.	Other:<br>- Sometimes, people over rely on science (or do not want to have the political decision and thus ask for scientific arguments to delay a process or to change the scope of a procedure/discussion)<br>- It should be considered among other things<br>- I do not know, the demand for HRA is so high that we have to do in-house or wait<br>- If standardized, it could really help in the process of obtaining environmental permits, it could be an additional help to the project planners	5	10.6	2	1	1

6. In which areas of decision-making are the different types of HRA results useful?

		All answers			
		Economy	Public health policies	Health protection actions	Other area (research, medicine)
a.	Number of diseases per e.g. 100000 persons	19	39	31	0
b.	Ratio of a disease occurrence between the exposed and non-exposed population	20	35	26	0
c.	Probability (e.g., 1:10,000) of developing a disease (i.e. getting ill) due to repeated (chronic) exposure to hazardous substances (e.g., at a work place)	21	35	35	2
d.	Number of newly diseased per e.g., 100,000 persons due to the exposure to hazardous substances (incidence)	21	33	32	1
e.	Increase of the number of diseased persons in a certain period among the entire population of interest (e.g., prevalence variation during selected time period)	18	32	29	1
f.	Number of diseased due to acute exposure to a hazardous (e.g., carcinogenic) substance	19	30	35	0
g.	Increase of the probability of disease occurrence due to specific exposure	18	26	28	1
h.	Additional healthcare costs due to exposure to hazardous substances	35	23	13	1
i.	Costs related to actions/measures needed for reducing the exposure	36	17	10	0

7. Is the comparison with standards, guidance values (GV), or reference values important when interpreting HRA results?

		All answers	All %	Group 1	Group 2	Group 3
a.	Yes	40	93.0	9	11	20
b.	No	3	7.0	0	2	1

8. If you answered "Yes" to the previous question, what actions are to be taken when the comparison indicates violation of standards, GV, or reference values?

		All answers	All %	Group 1	Group 2	Group 3
a.	Prohibition of the activities causing violation	17	37.8	1	6	10
b.	It is not clear what actions are to be taken	15	33.3	2	3	10
c.	Violation has no effect on the decision-making	3	6.7	1	0	2
d.	Other:<br>- Prohibition of the activities until the standards are further re-evaluated;<br>- I do not know;<br>- Guided prohibition of the activities causing violation;<br>- Mitigation of exposure (e.g. protective equipment for workers),<br>- Depends on the specific intent of the Guidance Value,<br>- Sometimes a closer examination of the standard, GV or reference value to determine if flexibility is warranted considering results that exceed those standards or reference values, specifically in a non-regulatory context.<br>- Research about the causes and actions needed for the situation;<br>- Prohibition or a limitation of the use of a substance which causes intolerable risks;<br>- Remediation	10	22.2	5	2	3

9. If HRA is a tool and the basis of taking specific decisions to reduce exposures, how do comparison with standards, GV, or reference values influence decision-making?

		All answers	All %	Group 1	Group 2	Group 3
a.	It improves the decisions, which would be taken based on HRA results	26	55.3	1	11	14
b.	In the case of violating standards, GV or reference values such comparison acts as a decision-making determinant: it overruns results of the HRA	10	21.3	2	2	6
c.	I do not know	7	14.9	4	0	3
d.	It is not clear how comparison with standards, GV or reference values affects decision-making	5	10.6	1	1	3
e.	If comparison with standards/GV shows no violation it has no role in decision-making	4	8.5	2	0	2
f.	Other:<br>- In the case of violations, the decision maker need to decide in "a direction" of better technology<br>- A risk matrix and risk treatment levels should also be a part of a risk management procedure	3	6.4	2	0	1

10. How would you rate the importance of the following elements of HRA?

Mean and standard deviation (St. dev.) are calculated by assigning the values of 5 for “Very important”, 3 for “Of medium importance” and 1 for “Less important”; “I do not know” answers were treated as NA and were not included in the calculation of the mean and standard deviation. Missing answers were also treated as NA.

	All answers				Mean	St. dev.
	Very important	Of medium importance	Less important	I do not know		
a. Vulnerability of the exposed population	39	6	2	0	4.57	1.02
b. Exposure	37	9	1	0	4.53	0.95
c. Magnitude of risk	37	9	0	1	4.61	0.80
d. Dose-response relationship	35	11	1	0	4.45	1.00
e. Risk characterization	33	14	0	0	4.40	0.92
f. Hazardous properties of materials	35	10	1	0	4.48	0.98
g. Assessment context of HRA (What is assessed and why)	26	17	3	1	4.00	1.25
h. Types of HRA results	22	20	4	1	3.78	1.30
i. Uncertainty of HRA	23	18	3	3	3.91	1.25
j. Coordination of HRA procedure	16	24	6	1	3.43	1.33
k. Participatory approach of HRA	15	20	9	3	3.27	1.47

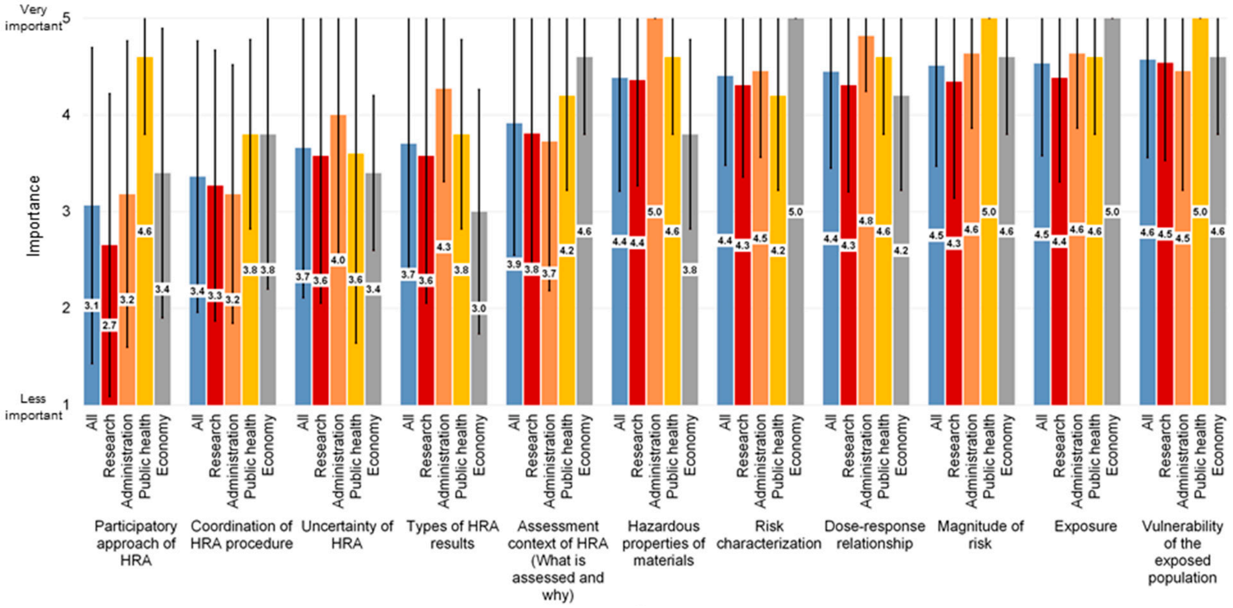

Figure S2: Perceived importance of HRA elements by the background of responders (all: n = 47, research: n = 26; administration: n = 11; public health: n = 5; economy: n = 5; showing mean values and standard deviations)

Comments: calculated standard deviations (shown as error bars on the Figure S2) indicate relatively large variation of responses.

11. In addition to HRA endpoints/results, how do the above-mentioned elements influence decisions?

	All answers	All %	Group 1	Group 2	Group 3
a. They contribute to the trustworthiness (credibility) of the HRA results and subsequent decisions	37	78.7	7	14	16
b. Depends on the decision-making case	14	29.8	3	1	10
c. They have no influence	0	0	0	0	0
d. Other:<br>- All the above-mentioned elements are important and necessary in HRA.<br>- They are necessary to understand risk and mitigate it.<br>- In industrial environments, an assessment precedes go/no go decisions.	3	6.4	1	0	2

12. Which elements of the HRA have had the strongest influence on your decisions so far?

Mean and standard deviation (St. dev.) are calculated by assigning the values of 5 for "Very important", 3 for "Of medium importance" and 1 for "Less important"; "I do not know" answers were treated as NA and were not included in the calculation of the mean and standard deviation. Missing answers were also treated as NA.

		All answers				Mean	St. dev.
		Influences the most	Has medium influence	Influences the least	I do not know		
a.	Exposure	30	11	0	0	4.46	0.90
b.	Magnitude of risk	29	11	0	2	4.45	0.90
c.	Vulnerability of the exposed population	28	11	1	1	4.35	1.05
d.	Hazardous properties of materials	28	9	2	2	4.33	1.15
e.	Dose-response relationship	24	15	3	0	4.00	1.27
f.	Risk characterization	20	16	4	1	3.80	1.34
g.	Assessment context of HRA (What is assessed and why)	20	15	5	1	3.75	1.41
h.	Uncertainty of HRA	10	27	3	1	3.35	1.10
i.	Types of HRA results	13	19	7	2	3.31	1.42
j.	Participatory approach of HRA	8	23	6	4	3.11	1.24
k.	Coordination of HRA procedure	5	20	15	1	2.50	1.34

13. Describe one or more examples of decision-making, where HRA results were, or could be, but were not, used successfully.

Research, toxicology and human health risk assessment (n = 11)

- In case of perfluorinated compounds
- No idea
- Salonit Anhovo (not used!)
- Lafarge, Kemis
- Lead and introduction of unleaded fuel
- Elimination of BPA from children's products, Dirty 12, phthalate restriction
- HRA could be used for microplastic or Wi-fi adverse effects, for example
- Uncontrolled use of pesticides and herbicides where studies have exhibited adverse outcomes to human health, though no strict policies have been activated
- Banning of lead (Pb) from consumer goods
- litigation support
- Mercury- looked at effect on children for cost benefit rather than cost of ALL health effects

Administration/regulation and emergency management (n = 6)

- Developing mitigation from risks presented by predestine exposure
- State regulation/standards of diesel exhaust
- PFAS
- In my work as a regulator/mitigator of potential risks from hazardous substances, I rely on risk assessments, I rely on risk assessments to quantify the potential risk and inform mitigation measures.
- In determining the risk of a release of air toxics in a particular neighborhood.
- Regular issuing of the approvals of the Chemicals office on the decisions about phytopharmaceuticals registration; approvals on the biocidal products; exposure of the inhabitants of Semič to PCB and OT

Public health (n = 3)

- Many underrepresented communities are studied...and there is no feedback or update. There is no remedy...There is no regard for the community.
- Environmental Impact Assessment (EIA) and Strategic Impact Assessment processes
- Neutrality

Industrial hygiene and job safety analysis (n = 3)

- So far, I have relied on deciding based on the comparison with limit values for chemical substances in the environment and emissions in workplaces. I see the usefulness of HRA when planning where to place new objects, which can have big impacts on the environment, as an additional element when judging whether to place such an object in the environment and as an element of directing the focus when planning on those parameters (emissions) that contribute the most to risk. It is definitely not the only decision-making factor. It is not only about making decisions about whether to place the object in the environment. More detailed knowledge about risks is an additional element, which can lead to the better planning of industrial objects.
- Remediation of a Superfund site.
- The current Corona virus pandemic.

14. How do you agree with the following statements concerning the HRA and Health Impact Assessment (HIA)?

Mean and standard deviation (St. dev.) are calculated by assigning the values of 5 for “I agree”, 3 for “Undecided”, and 1 for “I disagree”; “I do not know” answers were treated as NA and were not included in the calculations of the mean and standard deviation. Missing answers were also treated as NA.

		All answers				Mean	St. Dev.
		I agree	Undecided	I disagree	I do not know		
a.	HRA is useful/effective in decision-making about exposure reduction	22	3	1	10	4.62	0.98
b.	HIA is useful/effective in decision-making about exposure reduction	15	6	3	12	4.00	1.44
c.	HRA and HIA are different, particularly regarding presenting and interpreting probability of the occurrence of health effects due to specific exposures	14	6	3	13	3.96	1.46
d.	HRA can be a part of HIA	14	4	4	14	3.91	1.60
e.	HIA can be a part of HRA	13	2	7	14	3.55	1.87
f.	HRA is more comprehensive than HIA, since it comprehends toxicological issues more thoroughly	10	5	6	15	3.38	1.75
g.	HIA is more comprehensive than HRA, since it integrates health impacts with social and economic development aspects	7	7	8	14	2.91	1.69
h.	The content of HRA and HIA is the same, they are only termed differently	3	5	19	10	1.81	1.39

Comments: There was a relatively large proportion of “I do not know” answers. In addition, a lot of the responders did not provide answers to this question.

15. Please explain why it is, or why it is not necessary to differentiate between HRA and HIA.

Research, toxicology and human health risk assessment (n = 13)

- Don't know
- One can be part of the other
- HRA includes the worst-case scenario based on the toxicological data; HIA forecasts impacts ("reality assessment"), which include different scenarios
- I guess the health risk is not the same as the impact that something has on health. Risk = potential threat, impact = certain threat?. Don't know the exact definition though. Statement is based on the terminology.
- If I understood the concepts correctly, HIA is integrated on a larger scale since it uses social and economic aspects, while HRA is more comprehensive since it can be applied in individuals.
- Primarily, it's better to differentiate HRA and HIA. Their topics are specific, and in order to have a complete type of assessment, they have to be developed separately. But when all topics are developed in detail, they could be integrated with each other. In my opinion, to have a multi-comprehensive analysis, we need both of them. So, it is necessary to differentiate HRA & HIA.
- A risk is a potential impact and an impact already involves a health effect
- It is necessary to differentiate between HRA and HIA because they are aimed at different goals. With HRA, we derive the safe levels of exposure for humans, while with HIA, we determine the effects of the exposure in a target population.
- "Health Impact Assessment is used for the evaluation of proposed policies and the adverse outcomes that may pose to public health/health of population groups.
- On the contrary, Health Impact Assessment is used for the evaluation of the adverse outcomes that may be posed to public health/health of population groups by chemical substances."
- Regarding the previous knowledge in the matter, and which is the source of information, news agencies or scientific papers.
- I don't know
- I do not yet know how HIAs are done, so I can't say.

Administration/regulation and emergency management (n = 5)

- I am not familiar with what an HIA is. I have used HRAs to make risk mitigation decisions.
- It's necessary because the HRA can be a component of the HIA relating to toxic and adverse biophysical effects, while the HIA includes other social and environmental components.
- Unsure what HIA is
- I am not sure I understand the difference between these
- It's necessary so that the public understands what actions and assessments are being done and how they influence risk management decisions.

Public health (n = 3)

- Purpose of the study. Who is studied? Decision-making capabilities.
- Those are different concepts with different goals.
- I don't know the answer; however, I intend to understand by the end of this course.

Industrial hygiene and job safety analysis (n = 4)

- Human health risk assessments are not comprehensive and tend to focus on biophysical risks from exposure to hazardous substances. Results from a human health risk assessment can be used within an HIA to predict human health effects of specific exposures.
- I do not know.
- I am not familiar with the HIA.
- I do not know.

Usage of health risk assessment in decision-making: Questionnaire and results of the fourth target group

(n = 12)

1. What type of HRA results are the most useful in informing decision-making?

		Answers	%
a.	Probability of developing a disease or other harm to health due to specific exposure(s) to hazardous substance(s)	9	75.0
b.	Increase in the cases of a specific disease in a specified period of time or the number of newly diseased due to specific exposure to a hazardous substance (e.g. prevalence variation, incidence)	8	66.7
c.	Link between incidence and mortality for each disease, and/or QALYs	7	58.3
d.	Potential (probability) and scope of costs related to actions/measures needed for reducing the exposure	7	58.3
e.	Potential (probability) of additional healthcare costs due to increased rates of specific diseases after exposure to hazardous substances	6	50.0
f.	Ratio of a disease or other harm to health occurrence between the exposed and non-exposed population	4	33.3
g.	Other:<br>- Results that answer the questions that decision-makers are asking and should be asking, no generic answer possible since it all depends on the situation<br>- Confounding factors and other possible causes<br>- Uncertainty in each of the measures—quantitative<br>- Initial health condition of the at-risk population	4	33.3

Comments: Other responses indicate the importance of fitfulness for purpose.

2. In your experience in HRA and the quality of support that those HRA have given to decision-making, what assessment endpoints have been the most useful?

		Answers	%
a.	Collective risk of death (expectation of total deaths)	7	58.3
b.	Probability of individual exposure	6	50.0
c.	Individual risk of death	6	50.0
d.	Collective risk of disease	6	50.0
e.	Level of individual exposure	5	41.7
f.	QALYs (or equivalent)	4	33.3
g.	Individual risk of disease	3	25.0
h.	Toxicity index	1	8.3
i.	Hazard index or hazard quotient	0	0
j.	Other:<br>- Again, this depends on the decision context, what matters depends on who is asking, for some questions QALYs are helpful, for others they are irrelevant	1	8.3

Comments: Other response indicates the importance of acknowledging the decision context.

3. Besides type of HRA results and endpoints (see Q1 and Q2) how useful/effective are different elements of HRA in the process of informing decision-making?

(Use scale from 1 to 5, 1 being the least useful/effective and 5 being the most useful/effective)

		Answers					Mean	St. dev.
		1	2	3	4	5		
a.	Decision alternatives and/or options for mitigating/avoiding exposure	0	0	0	4	8	4.67	0.49
b.	Uncertainty of HRA results	0	0	0	6	6	4.50	0.52
c.	Transparency and clarity of the assessment process	0	0	0	6	6	4.50	0.52
d.	Assessment context of HRA	0	0	2	3	7	4.42	0.79
e.	Dose/exposure—response relationship	0	0	2	3	7	4.42	0.79
f.	Magnitude of risk, provided reference values in terms of probability and scope/intensity of health consequences	0	1	2	1	8	4.33	1.07
g.	Exposure sources	0	0	4	4	4	4.00	0.85
h.	Exposed population/individuals	0	0	3	7	2	3.92	0.67
i.	Exposure setting	0	0	4	5	3	3.92	0.79
j.	Exposure duration	0	0	5	3	4	3.92	0.90
k.	Exposure routes	0	1	5	2	4	3.75	1.06
l.	Stakeholder participation	0	0	6	4	2	3.67	0.78
m.	Reputation and credibility of the risk assessor	1	0	6	1	4	3.58	1.24
n.	Hazardous material pathway	0	1	4	7	0	3.50	0.67
o.	Hazardous properties of materials to which exposure takes place	1	2	3	4	2	3.33	1.23

p.	Costs and duration of HRA procedure	1	5	4	1	1	2.67	1.07

4. How does HRA actually influence the decision-making in the real world?

		Answers	%
a.	HRA and its results are used to improve a wide range of considerations of the decision-making process	8	66.7
b.	HRA results are one of many different important information used in decision-making; decision-makers explicitly put weights on these information and explain values behind	6	50.0
c.	I do not know	1	8.3
d.	HRA results according to the type of the assessment are direct and most important foundation of decision-making—decision-makers explicitly associate and justify their decisions with specific risk assessment results	0	0
e.	HRA results do not have real effect on decision-making, even if presented as important, since they are probabilistic in nature and are uncertain	0	0
f.	Other:<br>- Depends<br>- All or any of the above, or none at all, depending on circumstances	2	16.7

Comments: Other responses indicate the importance of acknowledging assessment (and decision) contexts.

5. How much do you agree with the following statements about actual uses of HRA in decision-making?

(Scale from 1 to 5; 1 strongly disagree, 5 strongly agree)

		Answers					Mean	St. dev.
		1	2	3	4	5		
a.	HRA results should be considered more often in decision-making	0	0	1	7	4	4.25	0.62
b.	Expectations and role of HRA results in decision-making are too high and irrational	2	4	3	3	0	2.58	1.08
c.	HRA unjustifiably prolongs the decision-making	7	2	3	0	0	1.67	0.89
d.	HRA results are not needed for decision-making	9	3	0	0	0	1.25	0.45

6. How does the comparison of hazard and exposure assessment results with guiding or reference values (e.g., Hazard Quotient - HQ, Hazard Index – HI, Risk Characterization Factor - RCF) influence the decision-making?

		Answers	%
a.	Such comparison is important for informing decision-making; however it should not be interpreted as risk assessment result since it does not provide information on probability/frequency of occurrence of specific health consequence due to specific exposure	5	41.7
b.	The comparison is not needed in addition to HRA results since it does not add to informing decisions and may actually mislead the decision toward radical attitudes	4	33.3
c.	I do not know	3	25.0
d.	Such comparison is a necessary part of risk characterization and can additionally improve the decisions	1	8.3
e.	When exceeding set values, such comparison acts as a decision-making determinant—it overruns other elements of risk characterization and leads to the reduction/avoidance of exposures (e.g. clean-up of contaminated sites, relocation or shutting down activities where hazardous substances are present, change of production or management process, change of nutrition habits, etc.)	1	8.3
f.	It is not clear how comparison with standards, guiding or reference values affects decision-making about actions to reduce exposures	1	8.3
g.	When below set values such comparison has no role in decision-making	0	0
h.	Other:<br>- Depends<br>- Depends on the basis of the standard	2	16.7

Comments: Other responses may indicate the importance of assessment context.

7. Probability and magnitude of health consequences are both inevitable components of HRA results. Please provide a brief summary on how each of them influence decision-making?

a) Probability:	b) Magnitude of risk:
Impact depends on combination of impact of risk and probability, high probability, and low impact (magnitude) can lead to the risk not being addressed properly.	Causes the "dread factor", which determines how unfavorable the outcome is and how important it is to prevent it from happening. Can lead to probability being discarded and contributes to the idea of a precautionary principle.
As it is an abstract concept, people have a harder time to understand what probability entails, making more room for cognitive biases. For example, most of the time policymakers focus on low probability risks, but with high consequences	Magnitude of risks is easier to be understood by people because it is possible to observe the effects and consequences of a certain risk, thus it carries more weight in the decision-making process
The human brain does not process probability information easily or accurately. We need improved ways of presenting and explaining it.	This is what the human brain likes to focus on and it can lead to poor decision-making

Depends on the context and whether the decision maker is focused on an individual or population	Depends on the context and whether the decision maker is focused on an individual or population
Risk is often defined, in various contexts, as probability times severity of injury. When this is the definition of risk, then probability is obviously important. However, probabilities are often highly uncertain, especially for rare or unfamiliar situations, in which case probability is not a useful concept. Alternatives include info-gap theory and its concept of robustness.	See part a)
I think you mean incidence.	I think you mean severity.
Determines the relative frequency of a disease	Determines the severity of the disease if an individual is affected
Probability is one component decision-makers should consider when weighing the risks.	Magnitude is another component that should be consider alongside other factors when weighing the risks.
Probability probably gets less attention than it should	More severe risks get more attention
Decision-makers are often uncomfortable with probability. Also hard to communicate that even a highly improbable event can occur and should not be taken as an error in the analysis	Magnitude of risk plays an important role - especially when benefit-cost analysis is required for decision
Depends on the risk attitude	Depends on the risk attitude and perception
Probability is a measure of how well an analyst understands the problem. E.g., as the variance of an analysis sample set decreases, an analyst's confidence may increase and time-to-decision may decrease.	Risk magnitude or impact is a more objectively measurable a quantity than is risk probability. As such, it tends to be the basis for prioritizing the array of risks under consideration.

Additional comments:

- I really did not understand the survey all that well. I found the questions somewhat opaque and ambiguously worded.
- Need to consider both incidence and severity. See Who 2014
- One issue missing is the dynamics of infections (incubation period, time of highest infection risk, latency periods).

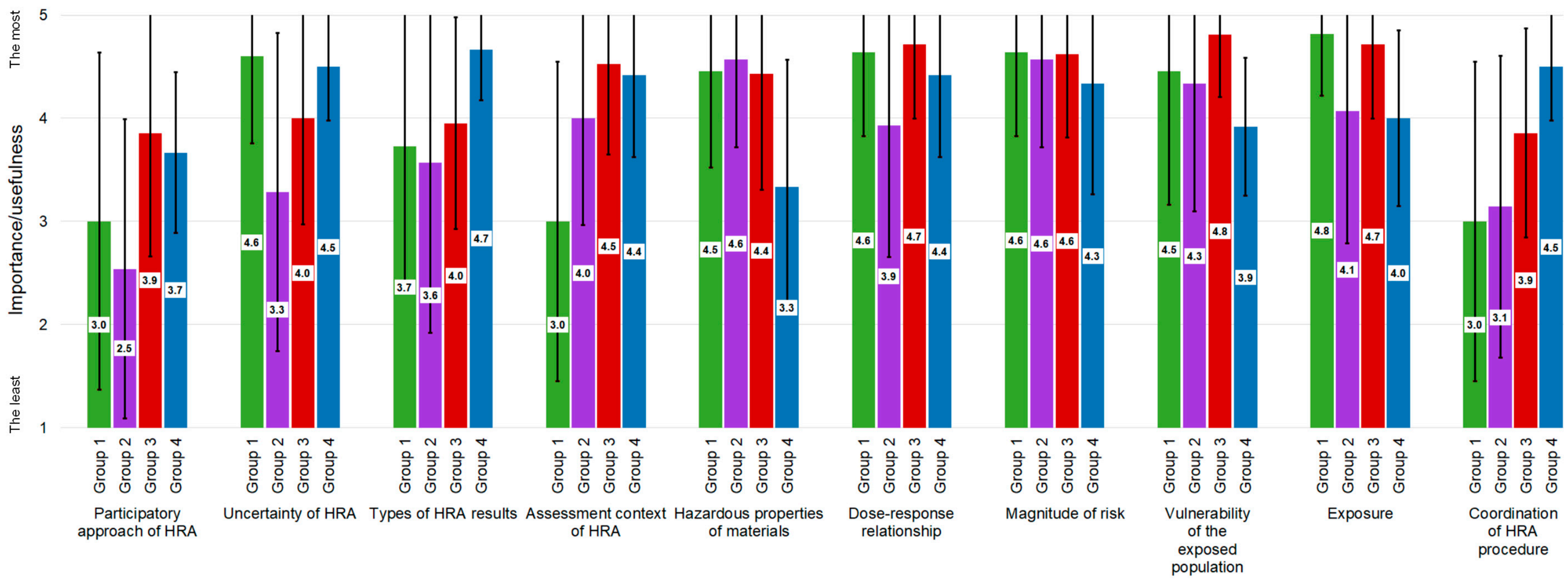

Figure S3: Perceived importance/usefulness of HRA elements for decision-making – comparison of responses of the four groups (showing mean values and standard deviations)

Comments: HRA element counted under "Types of HRA results" for group 4 was "Decision alternatives and/or options for mitigating/avoiding exposure"; "Exposed population/individuals" element of the second questionnaire (group 4) was counted under "Vulnerability of the exposed population"; "Transparency and clarity of the assessment process" element of the second questionnaire was counted under "Coordination of HRA procedure". Second questionnaire asked about several different elements of exposure assessment (i.e., exposure sources, exposure setting, exposure duration, exposure routes and hazardous material pathway). For comparison in Figure S3, we only included "Exposure sources" under "Exposure". Error bars showing calculated standard deviations indicate relatively large variation of responses.

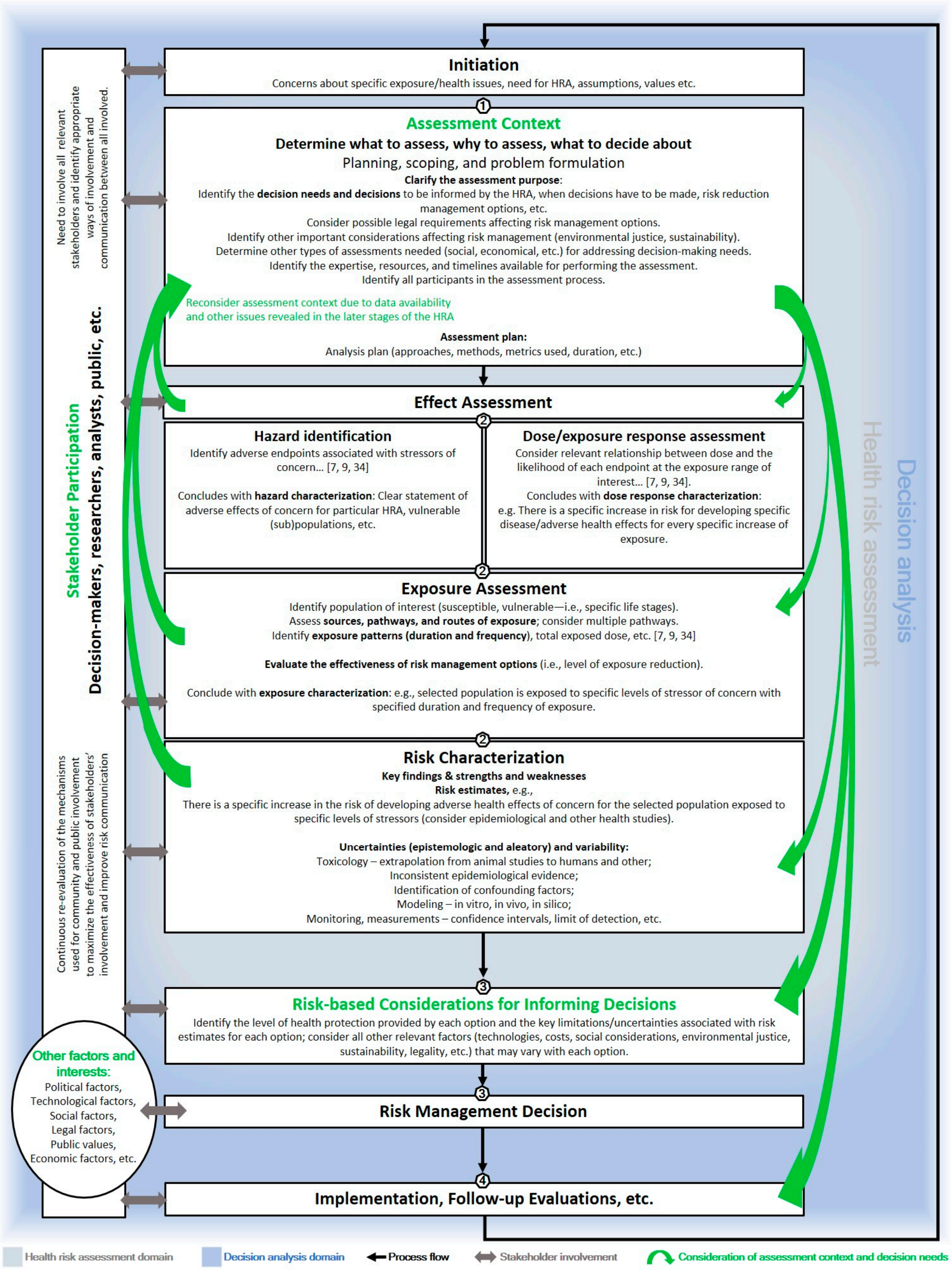

Figure S4: Opportunities for improving the utility of health risk assessment (expanded).
